# Suppression of cancer stemness by upregulating Ligand-of-Numb protein X1 in colorectal carcinoma

**DOI:** 10.1371/journal.pone.0188665

**Published:** 2017-11-30

**Authors:** Lin Ma, Lan Wang, Yating Shan, Muhammad Nafees, Elshoura Ihab, Ruhui Zhang, Fangjun Wang, Wu Yin

**Affiliations:** 1 State Key Lab of Pharmaceutical Biotechnology, College of life Sciences, Nanjing University, Nanjing, P. R., China; 2 Department of Respiratory Medicine, The Affiliated Jiangyin Hospital of Southeast University, Jiangyin, China; 3 State Key Laboratory of Coordination Chemistry, School of Chemistry and Chemical Engineering, Nanjing University, Nanjing, P. R., China; 4 Department of Gastroenterology, The Affiliated Jiangyin Hospital of Southeast University, Jiangyin, China; Università degli Studi della Campania "Luigi Vanvitelli", ITALY

## Abstract

Cancer stem-like cells (CSCs) have been reported to play major roles in tumorigenesis, tumor relapse, and metastasis after therapy against colorectal carcinoma (CRC). Therefore, identification of colorectal CSC regulators could provide promising targets for CRC. Ligand-of-Numb protein X1 (LNX1) is one E3 ubiquitin ligase which mediates the ubiquitination and degradation of Numb. Although several studies indicate LNX1 could be a potential suppressor of cancer diseases, the functions of LNX1 in mediating cancer stemness remain poorly understood. In this study, LNX1 was identified as a negative regulator of cancer stemness in CRC, which was downregulated in colonospheres or side population (SP) cells. Furthermore, the coxsackievirus and adenovirus receptor (CXADR) was found to be one critical downstream mediator of cancer stemness regulated by LNX1. Interestingly, the anti-breast cancer drug tamoxifen was found to be an agonist of LNX1 and suppress cancer stemness in CRC. In sum, this study provided the evidences that LNX1 signaling plays important roles in regulating the stemness of colon cancer cells.

## Introduction

As one of the most commonly diagnosed cancer diseases in both men and women, colorectal carcinoma (CRC) has caused serious concerns demographically and economically throughout the world. Statistically, there are 95,270 cases of CRC and 49,190 deaths in the US in 2016 whereas in China, almost 376,000 patients were diagnosed with CRC and 191,000 of both genders died of CRC in 2015 [1]. With the occurrence of chemoresistance and tumor relapse after therapy, the frontier of cancer stemness has become the focus of recent developments in CRC research [2,3]. Plethora of research reports have demonstrated that there is a small group of cells named as cancer stem-like cells (CSCs) possessing the ability of self-renewal and higher proliferation rate with increased capacity of invasion, metastasis and tumor formation [4,5,6]. Although there is no consensus on the concept on CSCs in academia, this group of cells are enriched in solid tumors following chemointervention and function as the “arch-criminal” which finally leads to drug resistance and tumor recurrence after therapy against CRC [7,8,9]. At present, targeting CSCs has become one of the promising strategies for the development of CRC therapy [10].

Identifying new properties of CSCs, exploring the biochemical mechanisms of CSCs and searching for the key regulator of cancer stemness will be instructive for the reversal of drug tolerance and the inhibition of the tumor recurrence mediated by cancer stemness in CRC study. Currently there are different methodologies to identify and isolate CSCs including cell sorting using the stemness-specific cell surface marker, detection of side population (SP) phenotype by Hoechst 33342 efflux, assessment of the ability to form spheres or tumors, and analysis of aldehyde dehydrogenase (ALDH) activity [11]. There are several CSC markers which have been identified in CRC study including CD133, CD44, ALDH1, LGR5, EpCAM, CD24, CD29, CD166, as well as ABC transporters [12,13,14,15,16,17,18]. As one broad-spectrum stemness marker, CD133 was widely used to identify the CSC population in various kinds of cancer cells including CRC [19,20,21,22]. Although there has been a rapid advancement in the field of CSCs research which have provided optimism for the application of more reliable CRC therapies, however, the identification of specific markers of colorectal CSCs still remains a challenge [23,24,25]. Besides the identification of CSC markers using antibodies, there are other ways to distinguish them in the heterogeneous solid tumor tissue. CSCs can be enriched in SP after fluorescence activated cell sorting due to ABC transporters such as ABCG2 activation in this population which cannot be stained with Hoechst 33342, compared with those treated with verapamil [26,27]. To evaluate the stemness, the extreme limited dilution assay (ELDA) has been widely used to determine the efficiency of sphere formation or tumor generation in nude mice. Briefly, serial dilutions of cells were cultured using serum-free culture methods to compare the rates of sphere formation between different groups [28]. Using this method, several CSC markers have been identified.

Ligand-of-Numb protein X1 (LNX1) is one E3 ubiquitin-protein ligase of NUMB which mediates the protein degradation of its downstream targets in a ubiquitin-dependent way [29]. There are two isoforms of LNX1 named respectively as LNX1 p70 and LNX1 p80, in which LNX1 p80 contains 4 PDZ domains at its C terminal and a RING domain at its N terminal that mediates ubiquitination and subsequent proteasomal degradation of its targets including NUMB [29]. In addition, LNX1 could independently interact with several other proteins via PDZ domains for its role as an E3 ubiquitin ligase [30,31,32,33,34]. Recently it has been demonstrated that members of the LNX family could be suppressor genes in various cancer diseases [33,35]. However, the precise function of LNX in mediated tumorigenesis or relapse after therapy is poorly understood. Here in this article, LNX1 was first identified as a negative regulator of cancer stemness in CRC and we showed that targeting LNX1 could provide a promising strategy against CSCs for clinical CRC research.

## Materials and methods

### Cell culture

Six colorectal carcinoma cell lines (Colo205, HCT116, HCT8, HT29, Caco-2 and LS174T) were purchased from American Type Culture Collection (ATCC; Manassas, VA, USA). HCT116, HCT8, HT29, Caco-2, LS174T were maintained in DMEM medium containing 10% fetal bovine serum (FBS) and 50 U/mL penicillin/streptomycin and Colo205 was cultured using RPMI1640 medium containing 10% FBS and 50 U/mL penicillin/streptomycin. The cell cultures were incubated at 37°C with a humidified atmosphere containing 5% CO_2_. The HT29 stable LNX1 knockdown cells line was created by lentiviral transduction of a pLentilox3.7 vector containing a specific construct (LNX1 shRNA sense 5’-GGAGAATGACCGTGTGTTA-3’). Another two stable LNX1-knockdown Colo205 cell lines were created respectively with two specific constructs targeting LNX1 including the above one and another construct (LNX1-2 shRNA sense 5’-GGTGCTTGTATAACTGTAA-3’).

### siRNA transfections

For transient knockdown studies, cells were transfected with pools of scrambled or target gene-specific siRNAs (100 nM) using Lipofectamine 2000 according to the manufacturer’s instructions. The sequences of designed siRNAs were as follows (sense): LNX1 5’-GGAGAAUGACCGUGUGUUA-3’, CXADR 5’-GGAAGUGACUUUAAGAUAA-3’, NUMB 5’- GGUUAGAAGAGGUGUCUAA-3’, c-Src 5’-GGCUCCAGAUUGUCAACAA-3’, ErbB2 5’-GGAAGGACAUCUUCCACAA-3’, Claudin1 5’-CAAUAGAAUCGUUCAAGAA-3’.

### Antibodies

Mouse monoclonal antibody against LNX1 was purchased from Abgent (San Diego, CA). Rabbit polyclonal antibodies against ALDH1A1, CD133, LGR5 and the mouse monoclonal antibody against ABCB5 were purchased from GeneTex (GeneTex, CA). Rabbit polyclonal antibody against CXADR was purchased from Novus Biologicals, Inc. (Littleton, CO). Anti-GAPDH and HRP-conjugated secondary IgG were purchased from Santa Cruz Biotechnology (Santa Cruz, CA).

### Sorting and analysis of SP cells

Cells were dissociated with trypsin, resuspended at 1×10^6^ cells per mL in DMEM with 2% FBS containing 5 μg/mL Hoechst33342 at 37°C for 90 min with or without 100 μM verapamil (Sigma) to inhibit ABC transporters. Then cells were incubated on ice for 10 min and washed with ice-cold PBS before flow cytometric sorting and analysis. Propidium iodide (PI) was used to distinguish live and dead cells and the Hoechst negative and PI negative population represents the group of SP. Flow cytometry data were analyzed using FlowJo 7.6.1 software.

### Colonosphere formation assay

Cells were seeded in ultra-low cluster plates (Corning Inc., Corning, NY) and cultured in DMEM/F12 serum-free medium (Invitrogen) supplemented with 20 ng/mL EGF, 20 ng/mL bFGF, 0.4% BSA, and 2% B27 (BD Pharmingen, Carlsbad, CA) as well as 1% methyl cellulose (Sigma-Aldrich). For ELDA, the cells at different densities were cultured 12 wells per cell density in stem cell medium in 96-well plates for 1 to 2 weeks. The numbers of wells with at least one tumorsphere (diameter >50 μm) were counted in a blinded manner. The frequency of sphere-initiating cells was calculated by ELDA online program at http://bioinf.wehi.edu.au/software/elda/.

### Tumorigenicity in vivo

Different numbers of HT29 cells were injected subcutaneously into the flanks of the male nude mice at three weeks of age which were housed in a specific pathogen-free facility. The tumors were counted after 18 days of injection and the tumor formation rate was calculated using ELDA analysis.

### Western blot

Cells were washed with 1x PBS and lysed on an ice with RIPA buffer supplemented with a protease inhibitor cocktail (P8340) (Sigma-Aldrich). Lysates were subjected to SDS/PAGE followed by blotting with the indicated antibodies. Signal detection was achieved using Clarity Western ECL substrate (Bio-Rad).

### RNA extraction and RT-PCR

Total RNAs were isolated with TRIZOL reagent (Invitrogen) following the manufacturer’s instructions. RT-PCR was performed in 20 μL of reaction mixture. PCR products were resolved on 1.2% agarose gels and stained with GelRed. Glyceraldehyde 3-phosphate dehydrogenase was also detected as a loading control.

### Statistics

Results are expressed as mean ± SD. Statistical analyses involving two groups were performed by means of Student’s t test. All data were processed with GraphPad Prism 5.0 software.

## Results

### LNX1 is a negative regulator of cancer stemness in colorectal carcinoma

**Fig 1:**
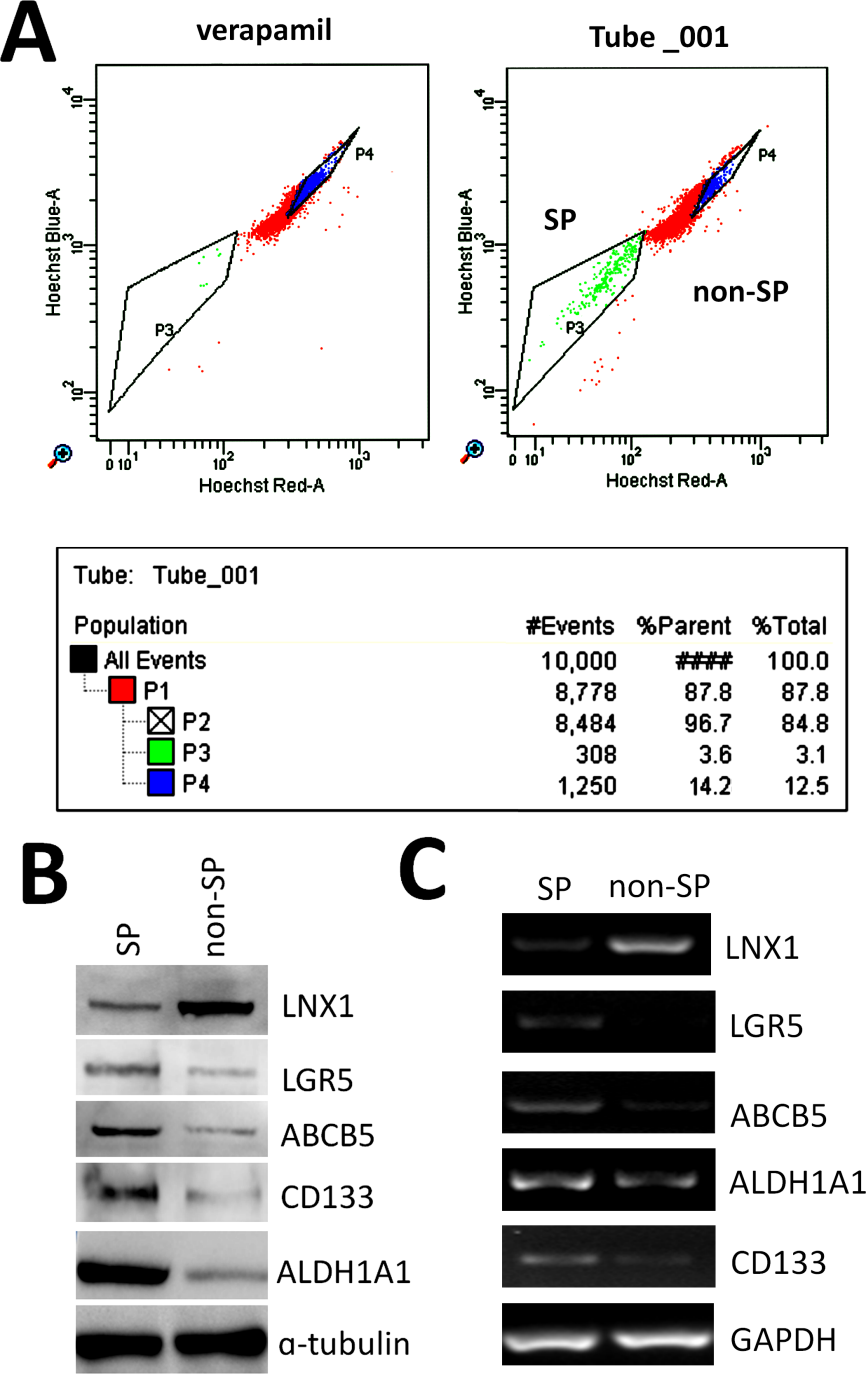
Analysis of LNX1 and CSC markers in CRC SP and non-SP cells. (A) Sorting of SP and non-SP cells using BD Aria software. The verapamil group was set as the negative control. (B) and (C) are the western blot and the semi-quantitative RT PCR analysis of genes (LNX1 as well as CSC markers in CRC including LGR5, CD133, ABCB5 and ALDH1A1).

**Fig 2:**
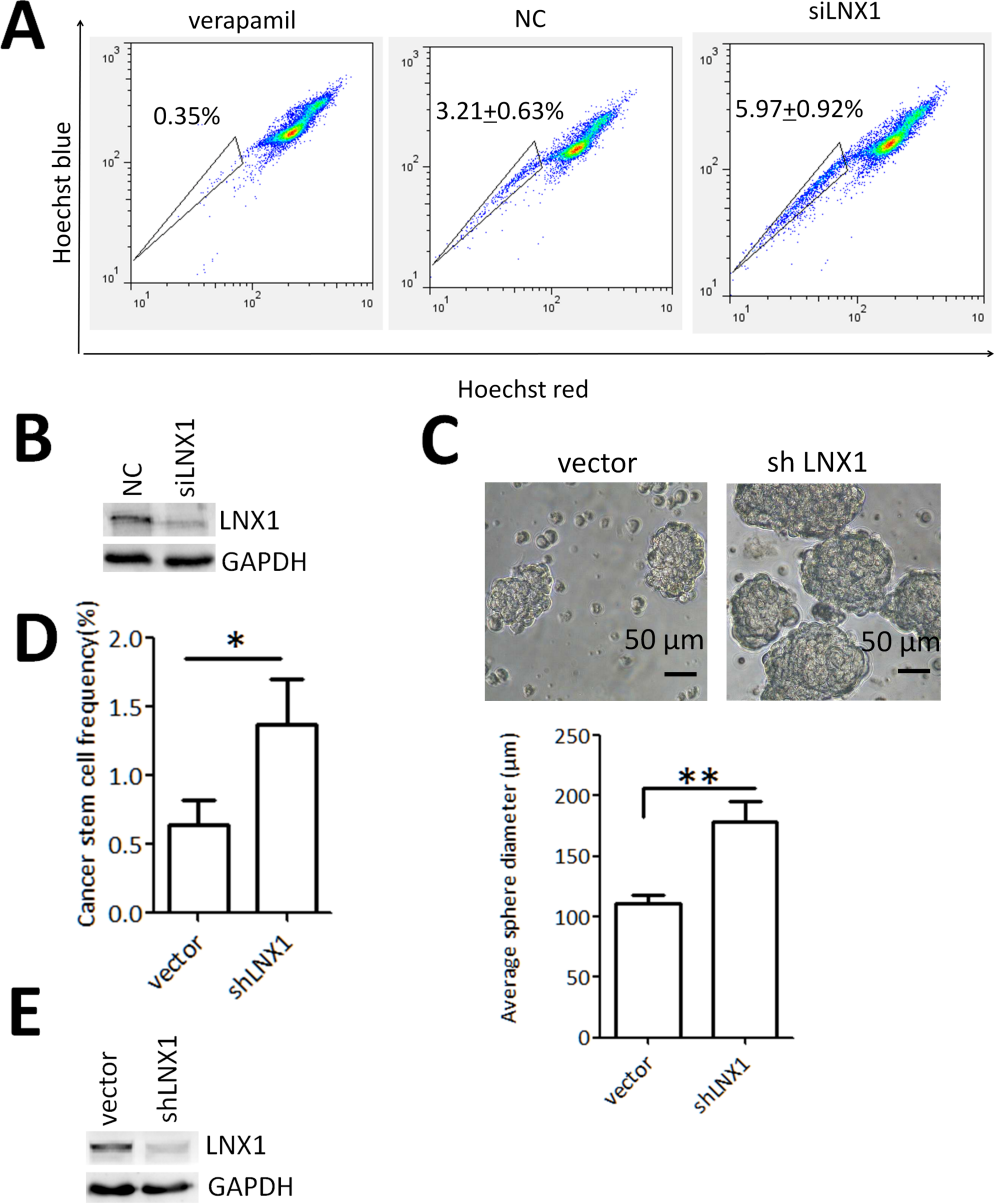
Analysis of the function of LNX1 in mediating cancer stemness in CRC. (A) Effect of LNX1 knockdown on the percentage of SP. (B) The efficiency of LNX1 knockdown using semi-quantitative RT PCR analysis. (C) Effect of LNX1 knockdown on the capacities of colonosphere formation (n = 8 per group). (D) Effect of LNX1 knockdown on the rates of colonosphere formation (p value was calculated using the online ELDA software). (E) The efficiency of LNX1 knockdown using shLNX1 lentivirus particles. Data from triplicates are presented as the mean±SD, *P<0.05, **P<0.01, ***P<0.001.

By detecting RNA level of LNX1 of the sorted SP and non-SP from the colorectal carcinoma cell line HT29, it was found that LNX1 was highly expressed in SP cells with higher levels of LGR5, ABCB5, ALDH1A1 and CD133 rather than non-SP group (Fig 1A, 1B and 1C). In an attempt to investigate the role of LNX1 in the SP maintenance, LNX1 knockdown assay using siRNA was performed revealing that downregulation of LNX1 increased the frequency of SP in HT29 (Fig 2A and 2B). To validate the function of LNX1 in regulating cancer stemness in colorectal carcinoma, the colonoshpere formation assay and ELDA analysis were performed using stable transfected shLNX1 HT29 cell line (vector as the control). Based on the observation, depletion of LNX1 enlarges the capacity of the colonosphere formation and increases the sphere formation rates (Fig 2C, 2D and 2E). Histological analysis was performed to confirm the origin of these xenograft tumors (S4 Fig). This observation was also confirmed by SP analysis and colonosphere formation assay using another colorectal carcinoma cell line Colo205 in which LNX1 was also depleted with two separate shRNA constructs using lentivirus infection (S1 Fig). Moreover, knockdown of LNX1 decreased the tumor formation rate in vivo (Table 1). Thus, LNX1 was identified as a negative regulator of cancer stemness in colorectal carcinoma.

**Table 1:** Effect of LNX1 knockdown on the tumor formation rate using the HT29 cell line.

HT29	Numbers of tumors/total injections				TCF(95% CI)	TCF Estimate	fold	P-value
	Cells per injection							
	1×10^6^	1×10^5^	1×10^4^	1×10^3^				
**vector**	**7/8**	**4/8**	**1/8**	**0/8**	**1/655511-1/127312**	**1/288919**	**1.0**	**P<0.05**
**shLNX1**	**8/8**	**6/8**	**3/8**	**0/8**	**1/115449-1/25115**	**1/53846**	**5.4**	

Different numbers of HT29 cells were injected subcutaneously into the male mude mice of 3 weeks old. Tumors were counted and the the formation rate was calculated using ELDA after 18 days of injection. TCF means tumor-initiating cell frequency. CI represents confidence interval.

### Inhibition of cancer stemness by LNX1 partially requires CXADR

**Fig 3:**
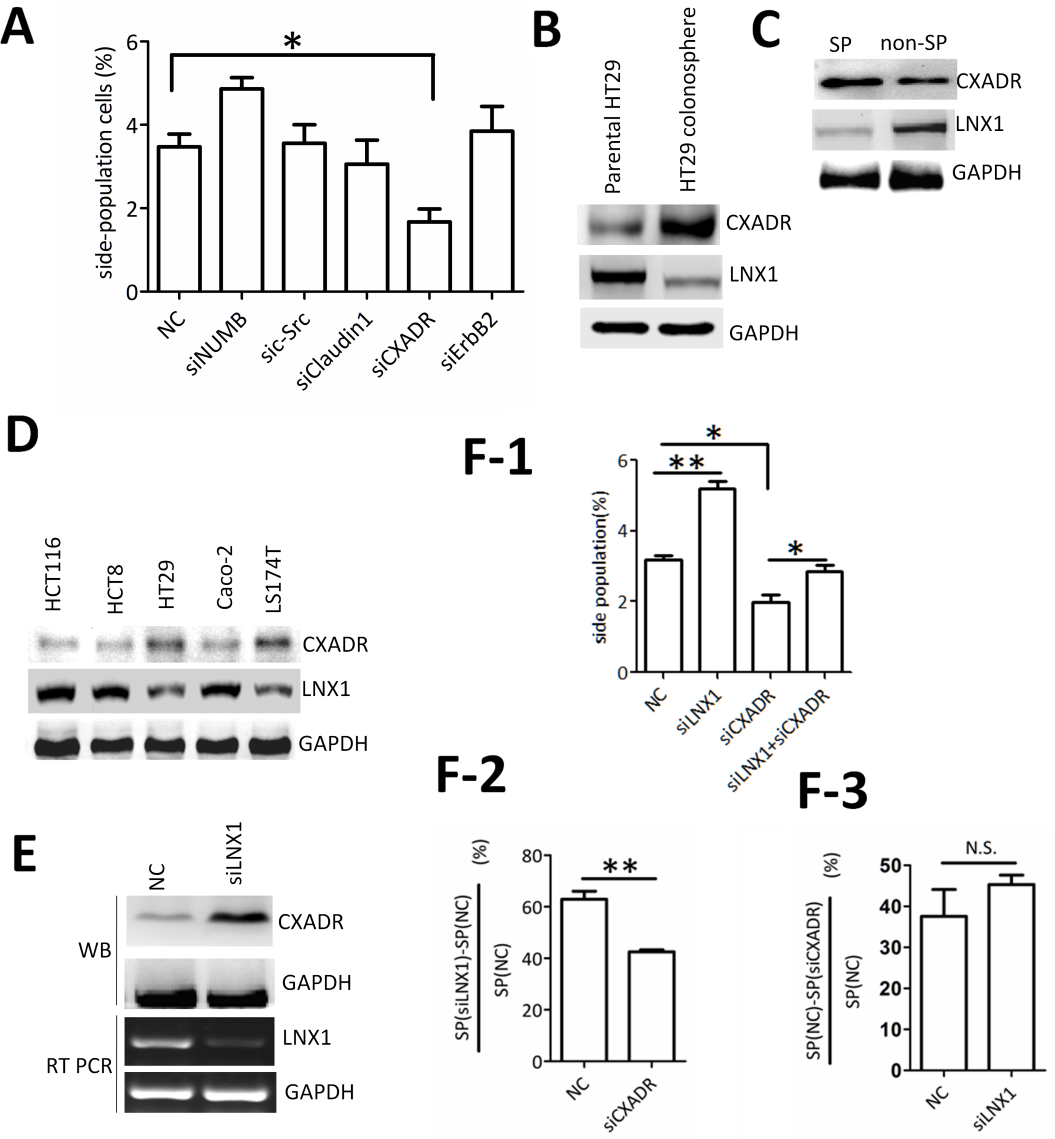
CXADR functions as a downstream CSC-meditator of LNX1. (A) Targets screening of LNX1 using SP analysis. B, C, D represent the expression analysis of CXADR and LNX1 respectively in HT29 compared with HT29-derived colonospheres (A); in SP and non-SP group(B); in various CRC cell lines(C).(E)Effect of LNX1 knockdown on CXADR level. (F-1) Effect of double knockdown of CXADR and LNX1 on the percentage of SP. (F-2) The inhibition percentage of SP frequency by LNX1 in the presence or absence of CXADR. (F-3) The inhibition percentage of SP frequency by CXADR knockdown in the presence or absence of LNX1. Data from triplicates are presented as the mean±SD, *P<0.05, **P<0.01, ***P<0.001.

To investigate the molecular mechanisms of the inhibition of cancer stemness mediated by LNX1, we screened potential substrates of LNX1 using RNAi and the SP analysis. Interestingly, knockdown of coxsackievirus and adenovirus receptor (CXADR) markedly decreased percentage of SP compared with other groups (Fig 3A and S2 Fig). Notably, CXADR was highly expressed in colonospheres of HT29 or SP cells rather than the control groups (Fig 3B and 3C). Besides, it was also observed that the level of CXADR was negatively correlated with the level of LNX1 in various colorectal carcinoma cell lines (Fig 3D).

In order to further confirm the relationship between LNX1 and CXADR, LNX1 knockdown assay was performed which reveals that depletion of LNX1 increased the level of CXADR (Fig 3E). Furthermore, LNX1 and CXADR double RNA interference assay showed that the upregulation of SP percentage in the absence of LNX1 was significantly affected by CXADR depletion. However, knockdown of LNX1 has little effect on the change of SP percentage caused by CXADR interference (Fig 3F–1, 3F-2, 3F-3 and S3 Fig). These results indicate that suppression of cancer stemness mediated by LNX1 partially requires the CXADR interference in colorectal carcinoma.

### Screening of LNX1 agonists to suppress cancer stemness in colorectal carcinoma

**Fig 4:**
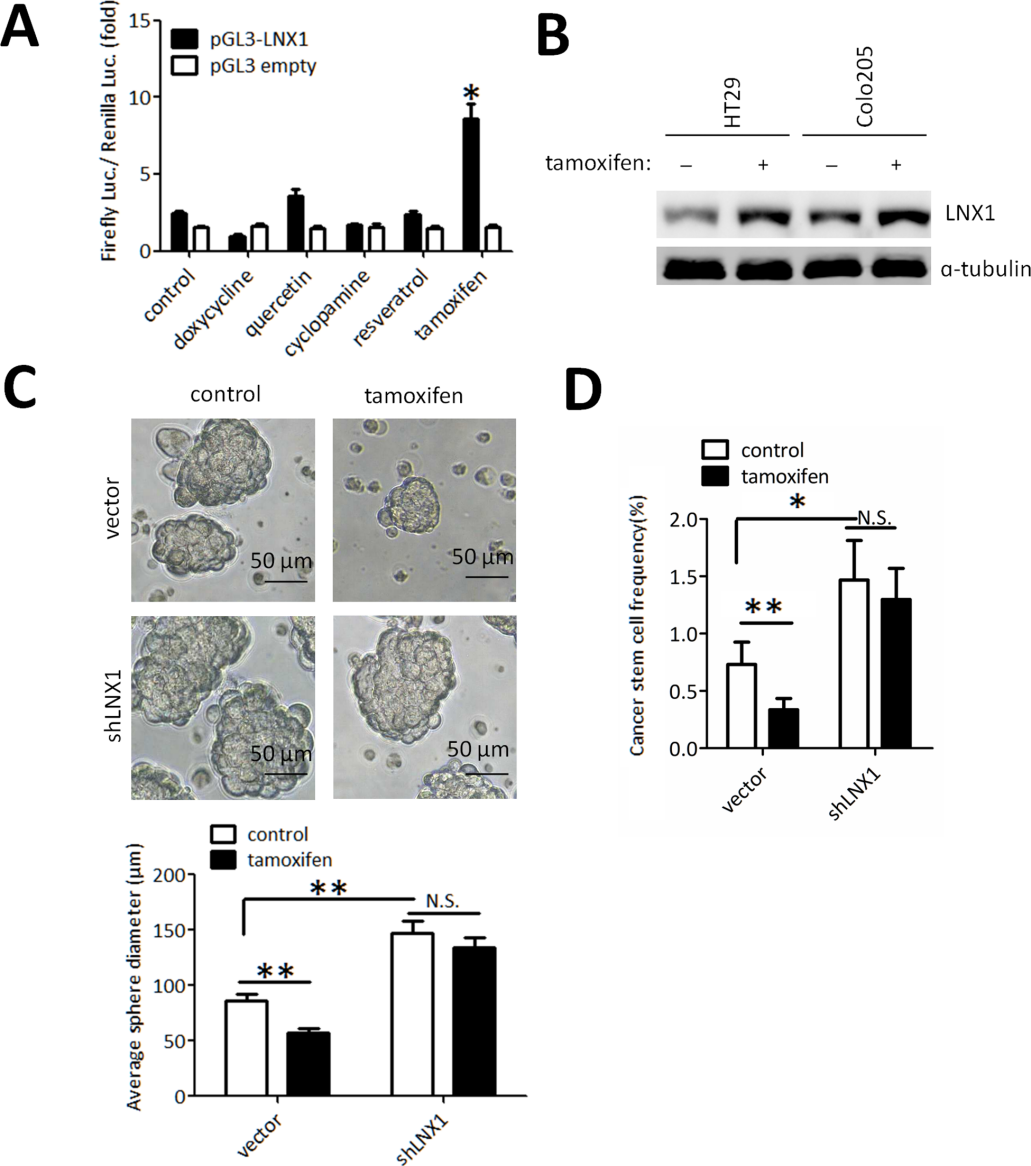
LNX1-based drug screening to suppress cancer stemness in CRC. (A) Effect on small molecules on LNX1 transcription. (B) Effect of tamoxifen on the LNX1 protein level in HT29 and Colo205 cell lines detected by western blot. (C)Effect of tamoxifen and LNX1 knockdown on the capacities of colonosphere formation (n = 8 per group). (D) Effect of tamoxifen and LNX1 knockdown on the rates of colonosphere formation (p value was calculated using the online ELDA software). Data from triplicates are presented as the mean±SD, *P<0.05, **P<0.01, ***P<0.001.

Quantitative LNX1 promoter activity profiles from HT29 cells treated with 5 drugs were obtained using the Dual Luciferase Reporter system, in which tamoxifen and quercetin both evidently increased the transcription activity of LNX1 (Fig 4A). Western blot results confirmed that tamoxifen could both upregulate the level of LNX1 in HT29 and Colo205 cell lines (Fig 4B). In addition, colonosphere formation assay and ELDA analysis reveal that tamoxifen could inhibit the capacity of colonosphere formation and decrease the sphere formation rates in the presence of LNX1, which demonstrated it as a potential target for colorectal carcinoma therapy against CSCs (Fig 4C and 4D).

## Discussion

Existence of CSCs is one driving force of colon tumorigenesis and malignancy and one major reason which leads to the drug resistance and tumor recurrence after therapy. There has been a rapid advancement in the field of CSCs research in colorectal carcinoma which has provided enough room for the application of more reliable cancer therapies [36]. Here we identified LNX1 as a new negative regulator of cancer stemness in colorectal carcinoma. It was also demonstrated that the expression of LNX1 was downregulated in colonospheres or SP of colorectal carcinoma cells. By exploring the underlying molecular mechanisms, it was found that LNX1 suppresses cancer stemness which partially requires the CXADR interference in colorectal carcinoma.

Several CSC markers on the cell surface functions as receptors to collect the information from the tumor microenvironment (TME) and facilitate the mutual communication with TME which was required by the tumor heterogeneity and the enrichment of CSCs [37,38,39]. Although CXADR has been reported to function as a virus receptor, its primary biological functions are unknown. Recently its has been shown that CXADR was highly expressed in tumor tissues rather than normal tissues and the anti-CXADR antibody could be a feasible drug candidate against cancer disease [40]. Here our results indicate that LNX1 suppresses cancer stemness which partially requires the downregulation of CXADR. As the component of the epithelial apical junction complex, CXADR protein was delicately regulated by the PDZ-domain-containing protein MAGI-1. Although LNX1 p80 contains both PDZ domains and the RING domain which is required for the ubiquitin-mediated protein degradation, however it cannot be ruled out the fact that CXADR was downregulated via other systems including the receptor endocytosis and degradation pathway rather than the LNX1-mediated ubiquitination pathway. Moreover, depletion of CXADR could not abolish the function of LNX1 in regulating cancer stemness, indicating there may be other downstream substrates of LNX1 in colorectal carcinoma.

**Fig 5:**
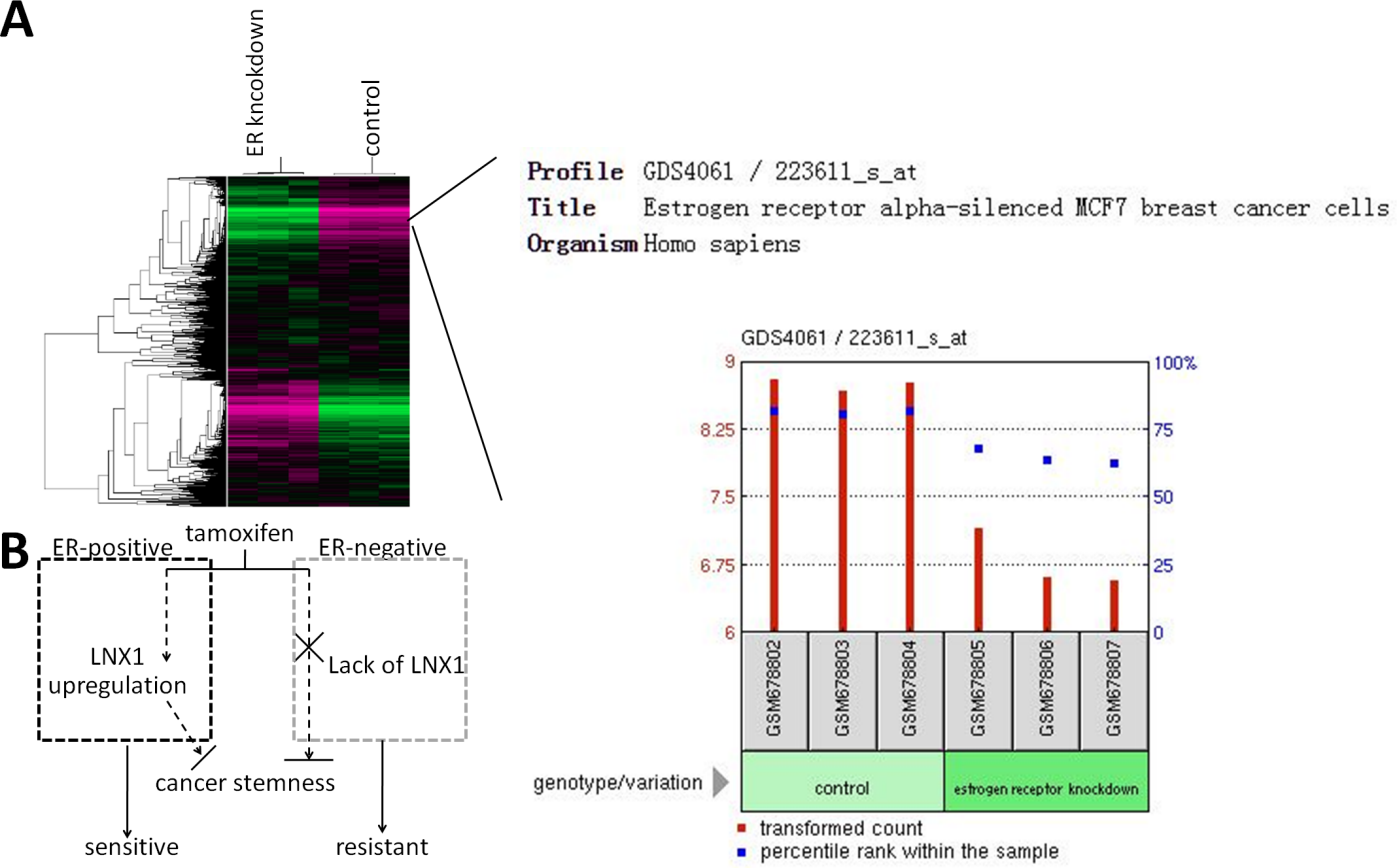
Probable mechanisms underlying tamoxifen therapy targeting ER-positive cells against breast cancer. (A) Effect of ER knockdown on the LNX1 level, data was obtained from the curated Datasets in the Gene Expression Omnibus (GEO) repository(GDS4061). (B) Schematic presentation of the predicted mechanisms underlying tamoxifen therapy targeting ER-positive cells against breast cancer, the broken lines indicate the possible cases at different circumstance. In ER-positive cells, tamoxifen could trigger the expression of LNX1 and exert its anti-tumor function, which could be abolished in ER-negative cells due to the restricted LNX1 level.

Based on the Dual-Luciferase Reporter system of LNX1 promoter, we screened different drugs in an attempt to find out a small molecule that could suppress cancer stemness via targeting LNX1. Interestingly, it was observed that tamoxifen downregulated the transcriptional activity of LNX1 and suppressed cancer stemness. Moreover, tamoxifen required LNX1 to downregulate the capacity and the rate of the colonosphere formation. Thus, LNX1 could be a potential drug target in cancer therapy against colorectal CSCs. As the endocrine agent most commonly used at all stages of breast cancer, tamoxifen has proved beneficial after therapy against estrogen receptor(ER)-positive breast cancer. Surprisingly, LNX1 was downregulated in ER-silenced MCF7 cells according to the gene expression profiles from curated Datasets in the Gene Expression Omnibus (GEO) repository (GDS4061). This phenotypic profiling using an Affymetrix Human Genome U133 plus 2.0 GeneChip is obtained by Sanaa Al Saleh using siRNA-mediated knockdown of the estrogen receptor α (ERα) in the breast cancer cell line MCF7 and it was observed that ERα knockdown resulted in estrogen/tamoxifen resistant cells with changed morphology, increased motility with the cytoskeleton rearrangement and the ability to invade simulated components of the extracellular matrix [41]. By exporting the GEO profile of LNX1, it was observed that LNX1 was downregulated in ERα-silenced breast cancer cells (Fig 5A). It might give a reasonable explanation why chemotherapy with tamoxifen has better effect on patients with ER-positive breast cancer than patients with ER-negative breast cancer. That is, LNX1 could not be significantly upregulated by tamoxifen in the absence of ER in breast cancer (Fig 5B). However, the relationship between LNX1 and ER still remains to be investigated.

## Supporting information

S1 Fig Analysis of the function of LNX1 in mediating cancer stemness in colorectal carcinoma cell line Colo205. (A) Effect of LNX1 knockdown on the percentage of SP in Colo205. (B) Effect of LNX1 knockdown on the capacities of colonosphere formation (n = 8 per group). (C) Effect of LNX1 knockdown on the rates of colonosphere formation (p value was calculated using the online ELDA software). (D) The efficiency of LNX1 knockdown using two shLNX1 constructs. Data from triplicates are presented as the mean±SD, *P<0.05, **P<0.01, ***P<0.001.
(TIF)

S2 Fig Screening of downstream targets of LNX1. SP analysis was performed using BD Aria software and was analyzed using FlowJo 7.6.1 software.
(TIF)

S3 Fig Effect of double knockdown of CXADR and LNX1 on the percentage of SP. SP analysis was performed using BD Aria software and was analyzed using FlowJo 7.6.1 software.
(TIF)

S4 Fig Histological analysis of HT29 xenograft tumors. Tumor sections were stained with hematoxylin and eosin (H & E).
(TIF)
