# Supplementary figures and images for: Suppression of cancer stemness by upregulating Ligand-of-Numb protein X1 in colorectal carcinoma
Source: PLoS One. 2017 Nov 30;12(11):e0188665. doi: 10.1371/journal.pone.0188665 (PMC5708683; doi:10.1371/journal.pone.0188665)

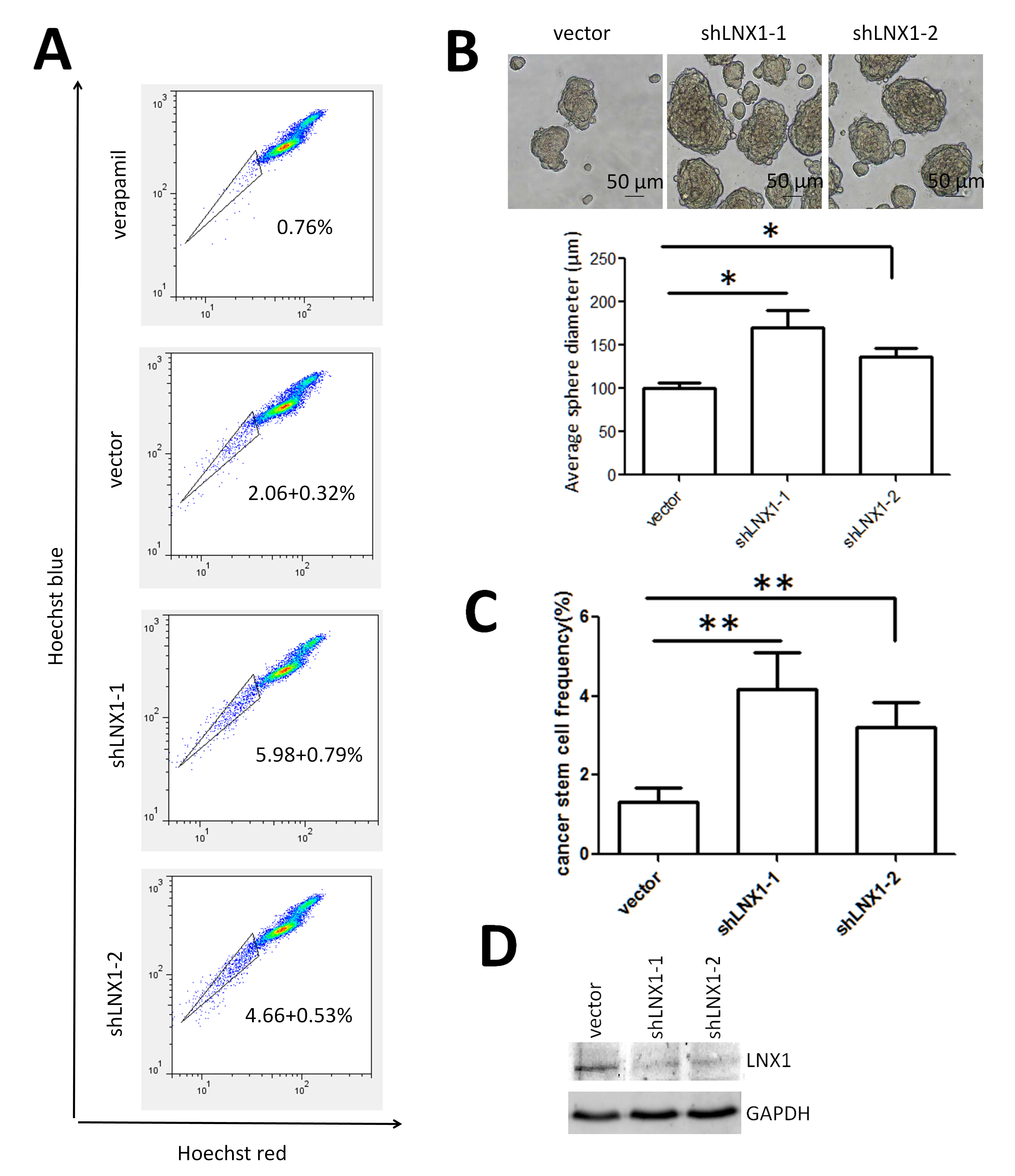

Supplement: S1 Fig — (A) Effect of LNX1 knockdown on the percentage of SP in Colo205. (B) Effect of LNX1 knockdown on the capacities of colonosphere formation (n = 8 per group). (C) Effect of LNX1 knockdown on the rates of colonosphere formation (p value was calculated using the online ELDA software). (D) The efficiency of LNX1 knockdown using two shLNX1 constructs. Data from triplicates are presented as the mean±SD, *P<0.05, **P<0.01, ***P<0.001. (TIF) [file pone.0188665.s001.tif]

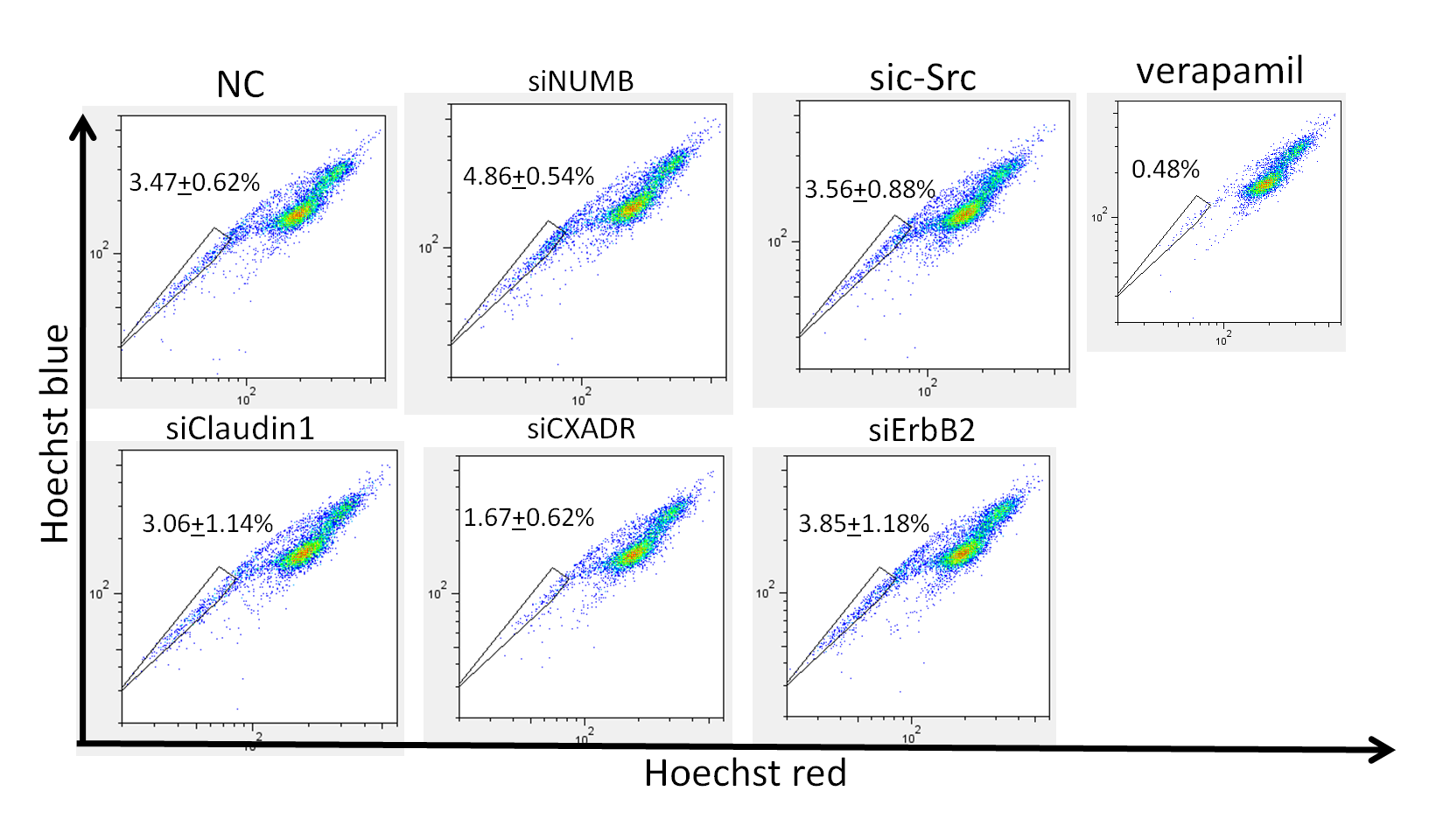

Supplement: S2 Fig — SP analysis was performed using BD Aria software and was analyzed using FlowJo 7.6.1 software. (TIF) [file pone.0188665.s002.tif]

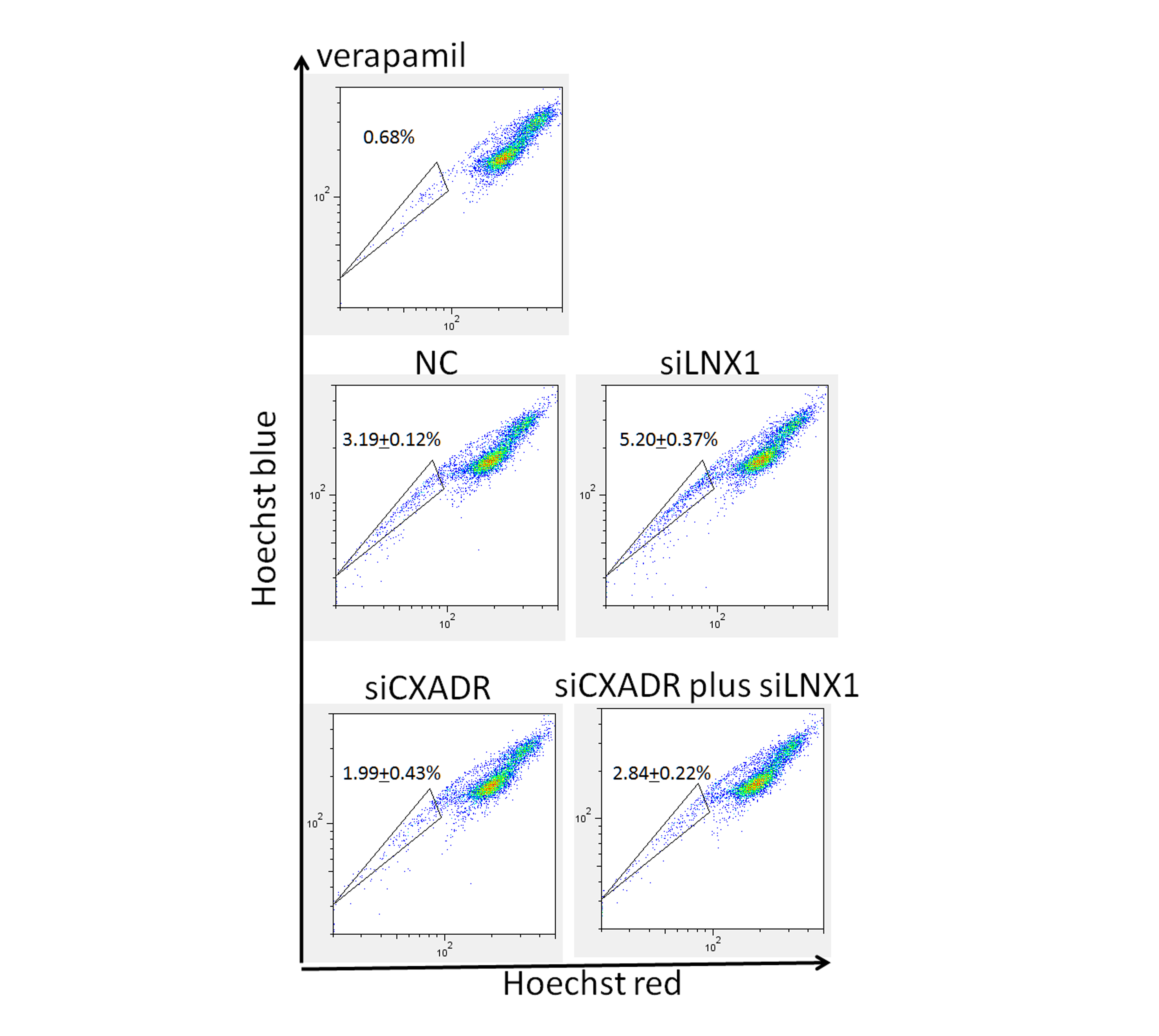

Supplement: S3 Fig — SP analysis was performed using BD Aria software and was analyzed using FlowJo 7.6.1 software. (TIF) [file pone.0188665.s003.tif]

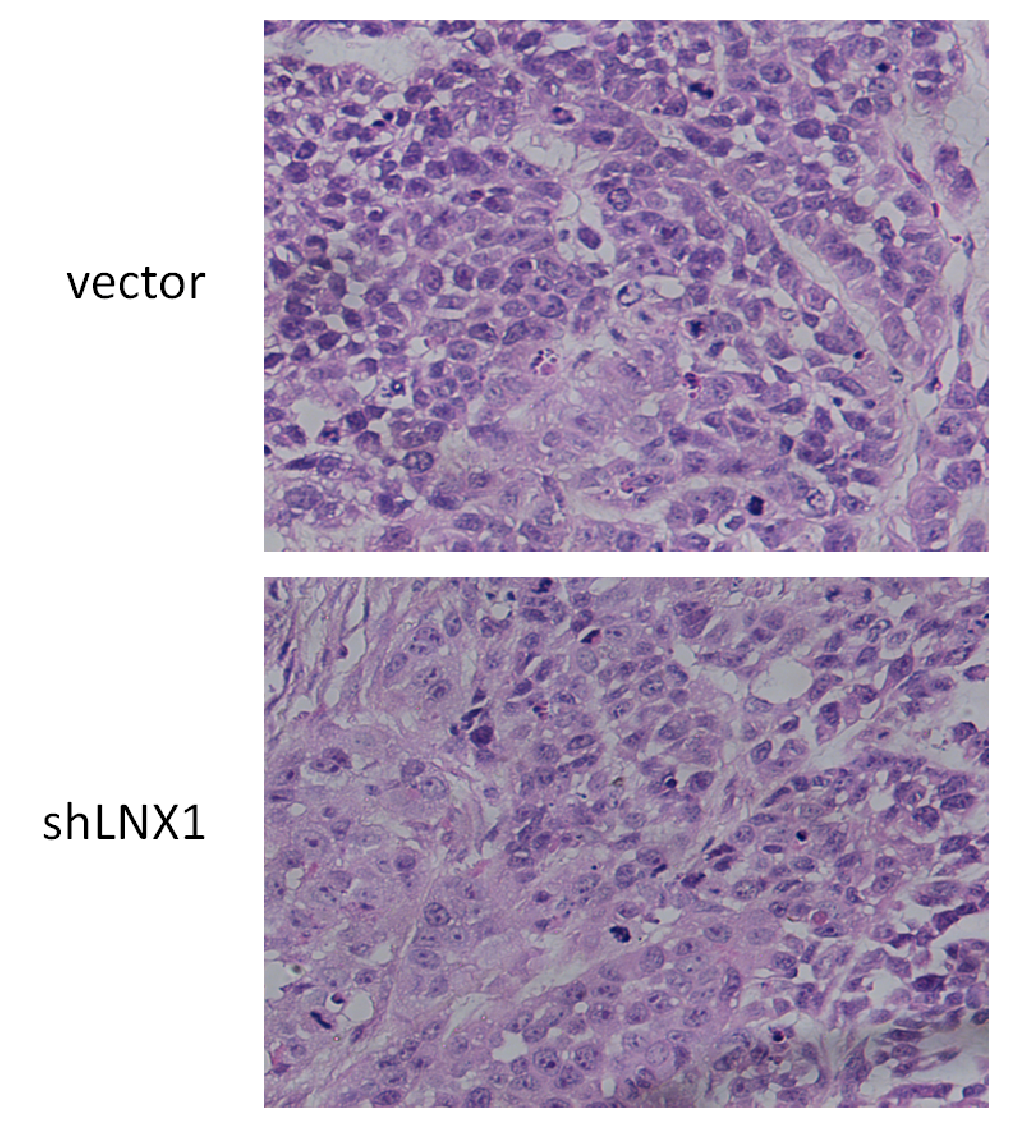

Supplement: S4 Fig — Tumor sections were stained with hematoxylin and eosin (H & E). (TIF) [file pone.0188665.s004.tif]
